# Supplementary material for: Isolation and Identification of Herbicidal Active Compounds from Brassica oleracea L. and Exploration of the Binding Sites of Brassicanate A Sulfoxide
Source: Plants (Basel). 2023 Jul 7;12(13):2576. doi: 10.3390/plants12132576 (PMC10346607; doi:10.3390/plants12132576)
Supplement: Supplementary file 1 [file plants-12-02576-s001.zip › plants-2485477-Supplementary Materials.pdf]

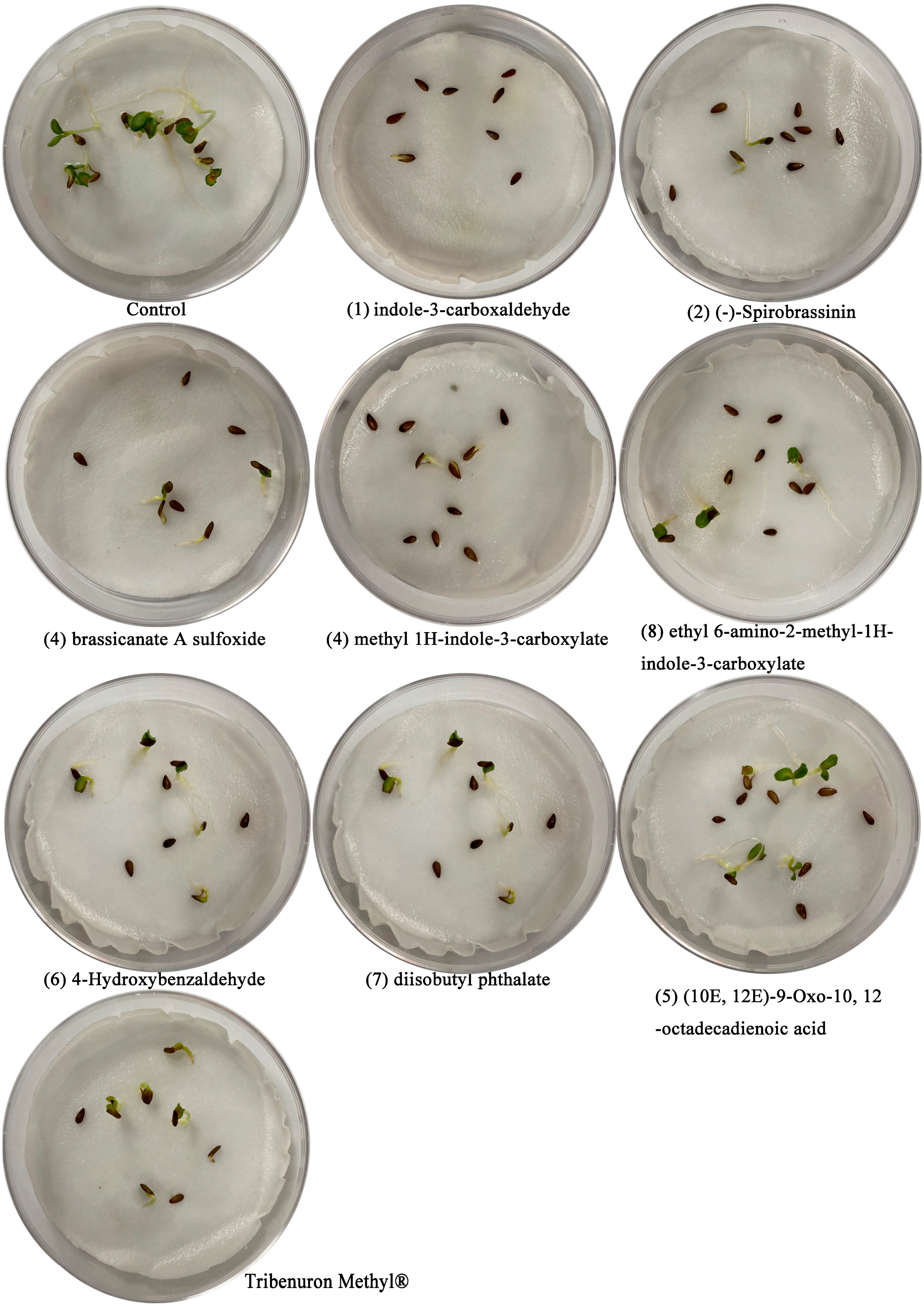

Figure S1. The effects of compounds from *B. oleracea* L. (1-8) and Tribenuron Methyl® on *L. sativa* Linn. germination and seedling growth.

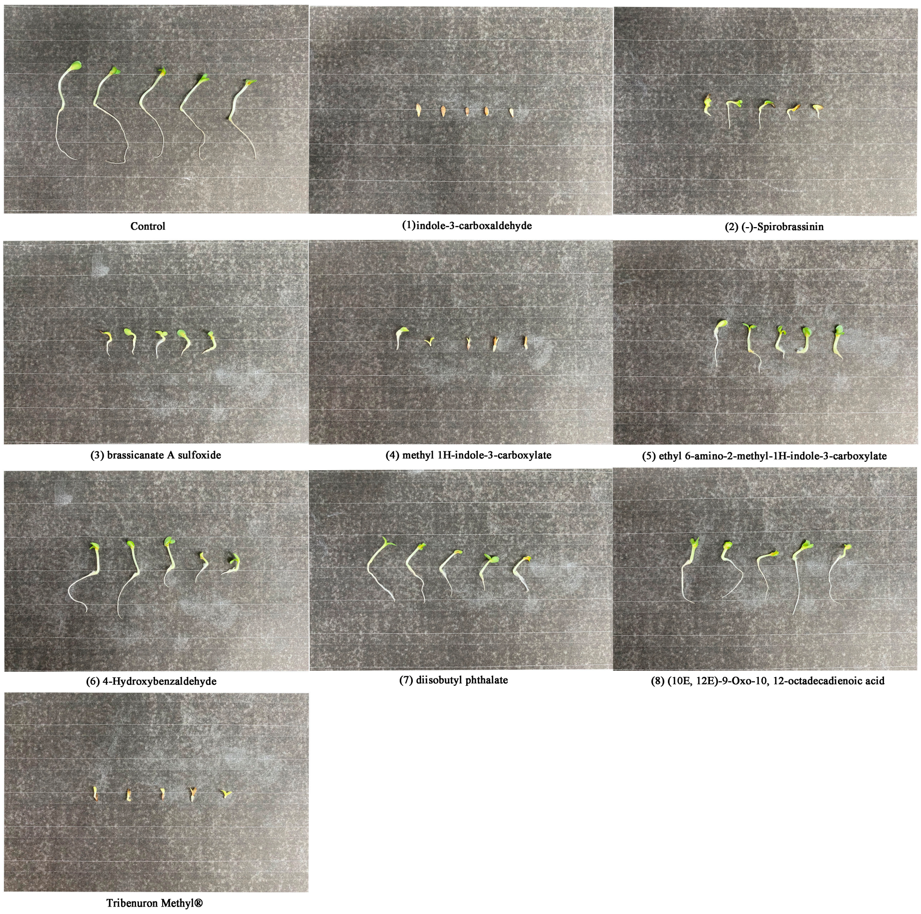

Figure S2. The effects of compounds from *B. oleracea* L. (1-8) and Tribenuron Methyl® on *L. sativa* Linn. seedling growth.

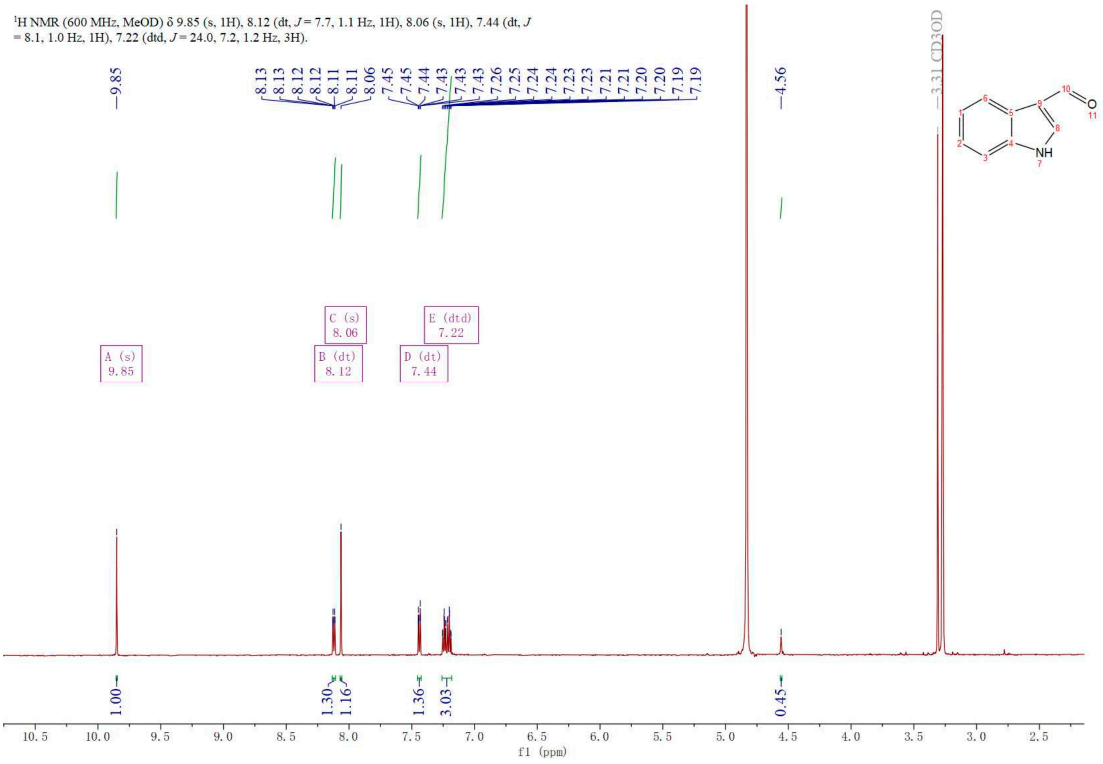

Figure S3. ^{1}H NMR of compound 1

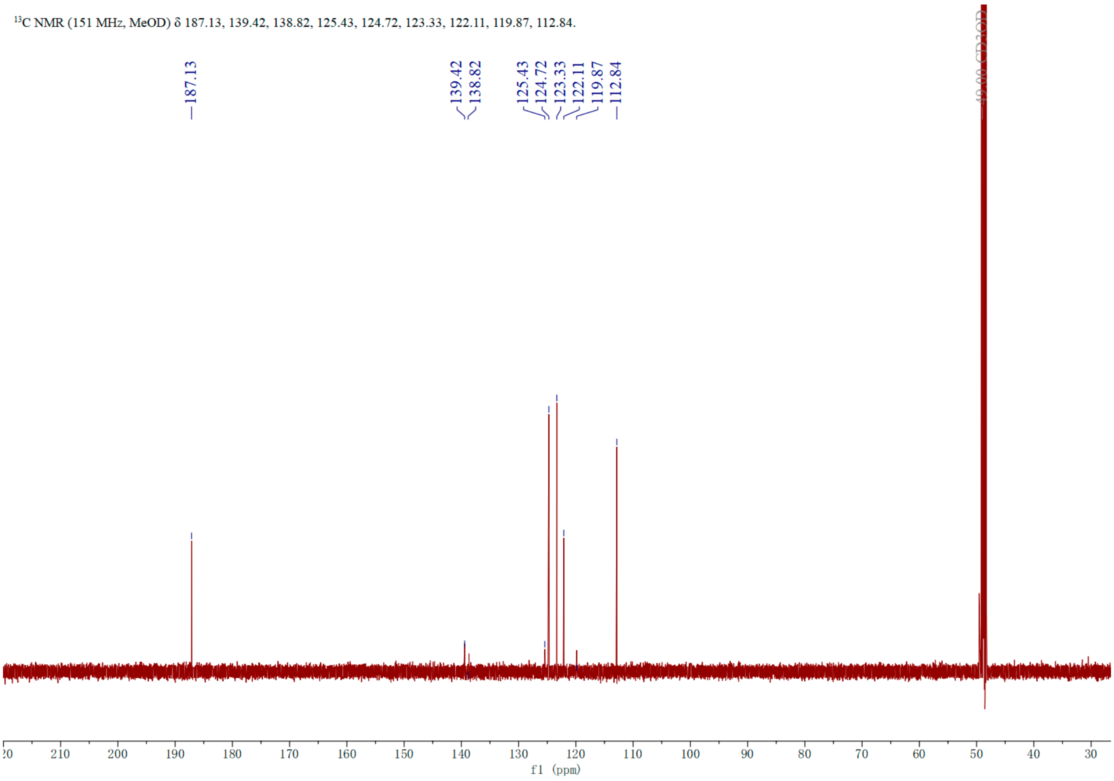

Figure S4. ^{13}C NMR of compound 1

^{1}H NMR (600 MHz, MeOD) δ 7.32 (d, J = 8.3 Hz, 1H), 7.27 (td, J = 7.7, 1.3 Hz, 1H), 7.07 (td, J = 7.6, 1.1 Hz, 1H), 6.90 (d, J = 7.8 Hz, 1H), 4.56 (d, J = 15.3 Hz, 1H), 4.46 (d, J = 15.3 Hz, 1H), 2.62 (s, 3H).

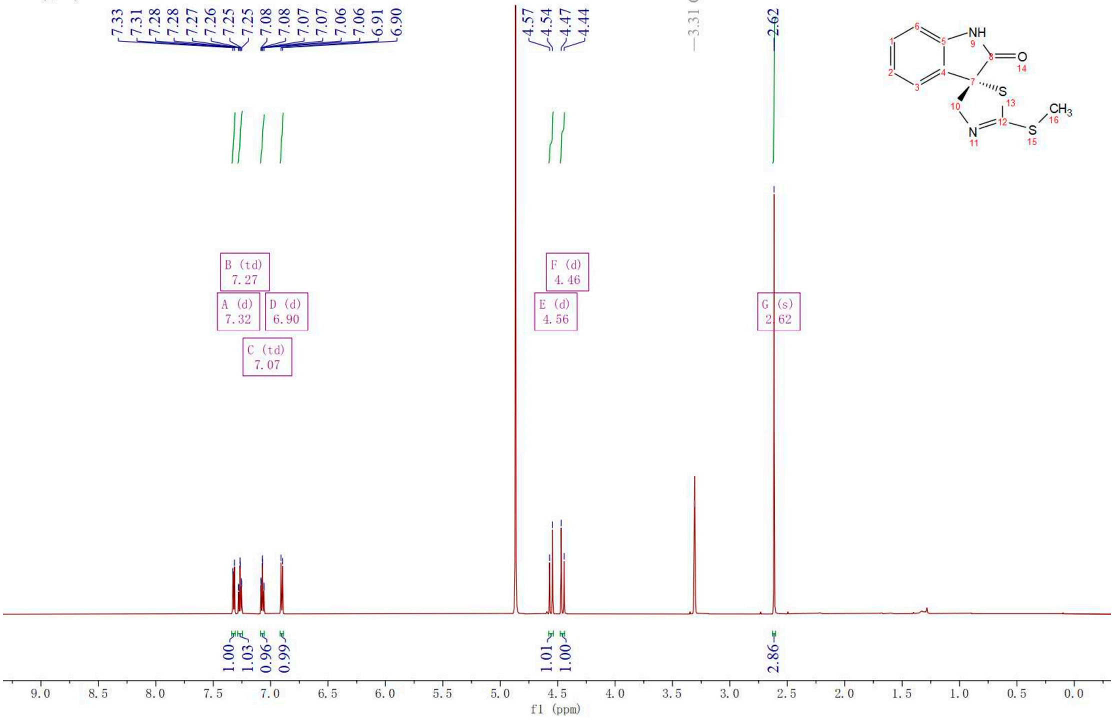

Figure S5. ^{1}H NMR of compound 2

^{13}C NMR (600 MHz, MeOD) δ 179.56, 166.37, 141.91, 132.00, 130.77, 125.09, 124.25, 111.26, 75.40, 65.75, 15.56.

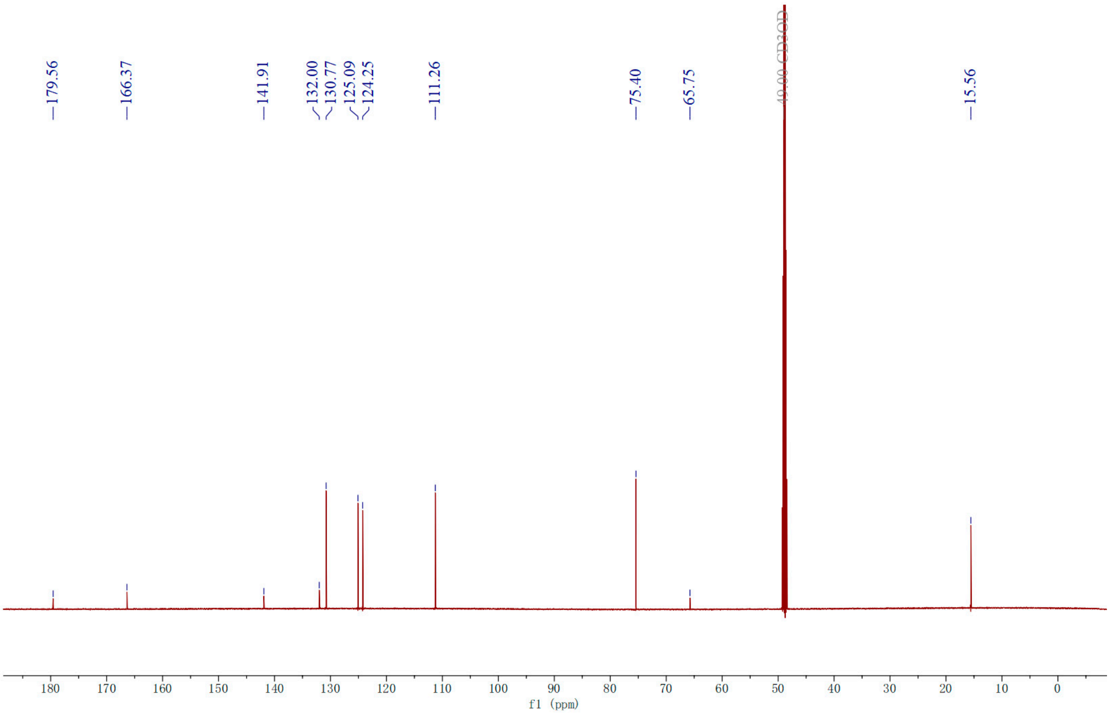

Figure S6. ^{13}C NMR of compound 2

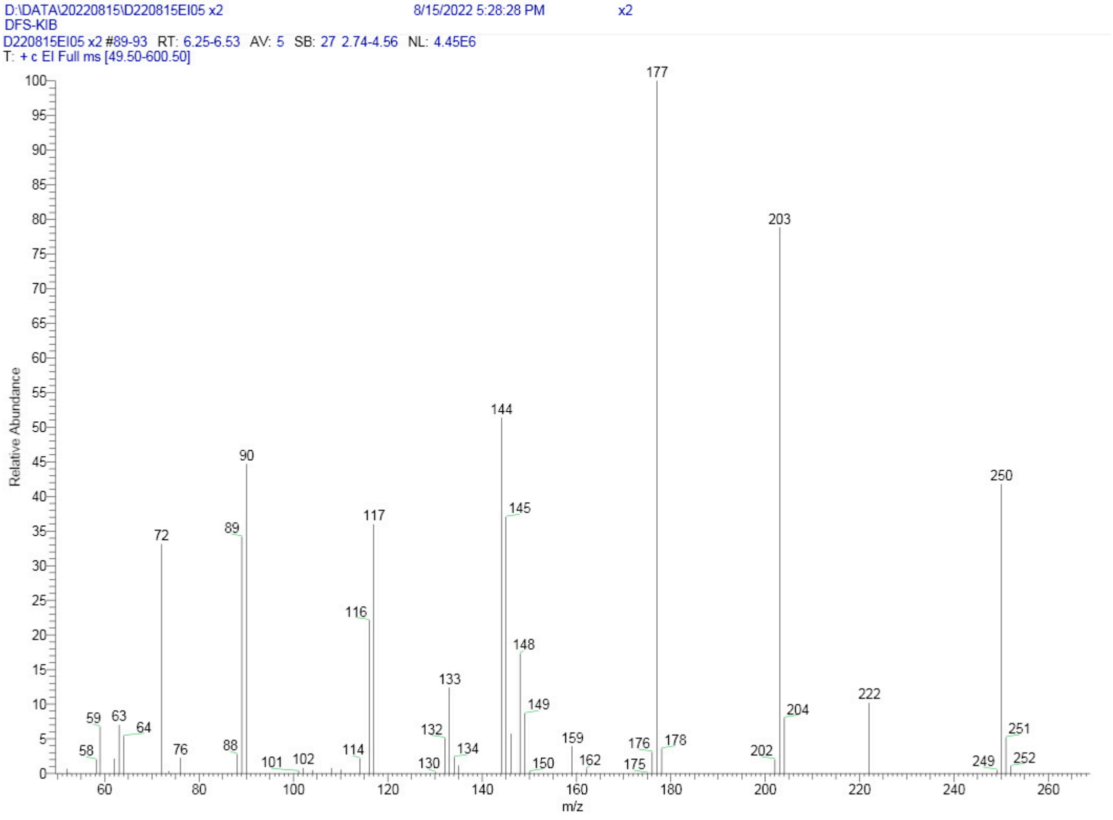

Figure S7. EI-MS of compound 2

Rudolph Research Analytical

This sample was measured on an Autopol VI, Serial #91058
Manufactured by Rudolph Research Analytical, Hackettstown, NJ, USA.

Measurement Date : Tuesday, 16-AUG-2022

Set Temperature : 25.0

Time Delay : Disabled

Delay between Measurement : Disabled

n	Average	Std.Dev.	% RSD	Maximum	Minimum
5	-68.42	0.35	-0.51	-67.92	-68.75

S.No	Sample ID	Time	Result	Scale	OR °Arc	WLG.nm	Lg.mm	Conc.g/100ml	Temp.
1	X2	06:39:24 PM	-68.75	SR	-0.330	589	100.00	0.480	24.9
2	X2	06:39:30 PM	-68.75	SR	-0.330	589	100.00	0.480	24.9
3	X2	06:39:36 PM	-68.33	SR	-0.328	589	100.00	0.480	24.9
4	X2	06:39:42 PM	-68.33	SR	-0.328	589	100.00	0.480	24.9
5	X2	06:39:49 PM	-67.92	SR	-0.326	589	100.00	0.480	24.9

Page 1 of 1

Figure S8. specific rotation of compound 2

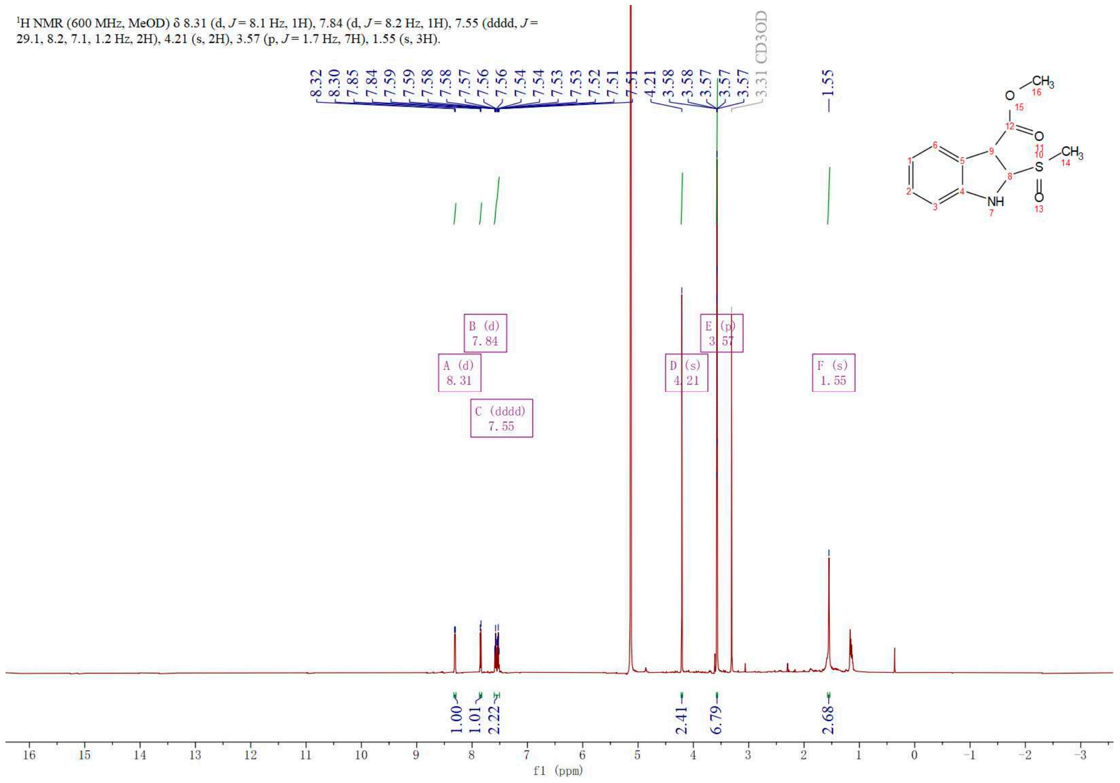

Figure S9. ^{1}H NMR of compound 3

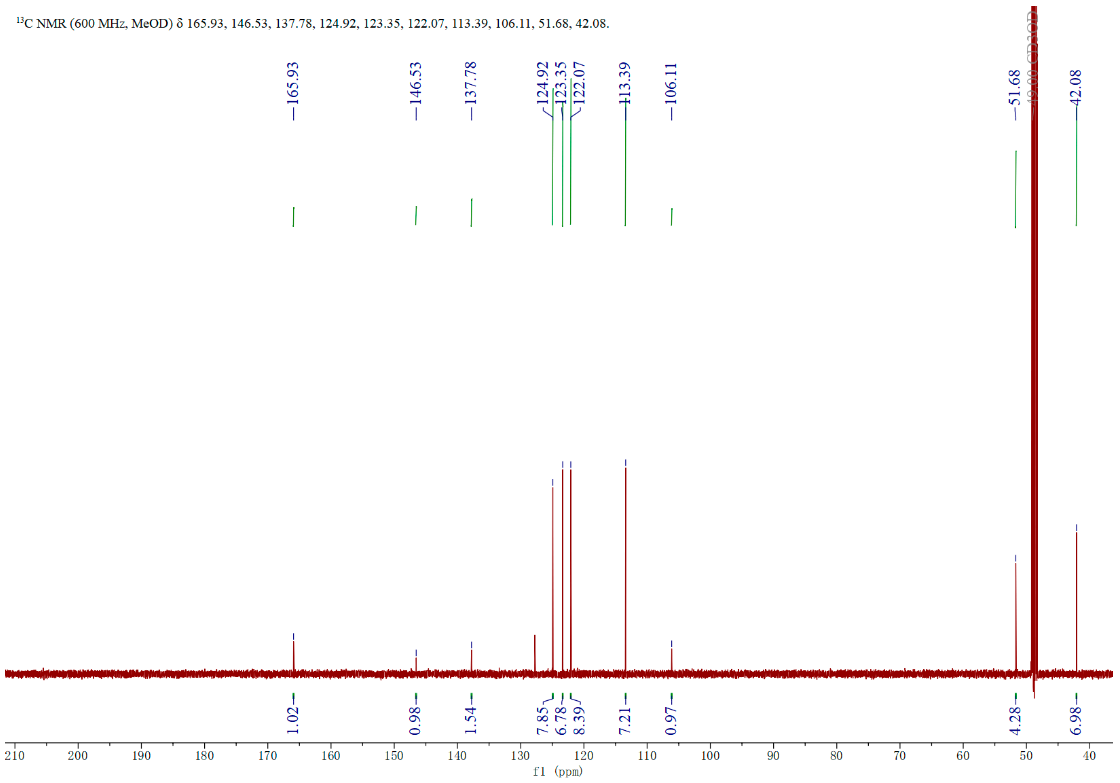

Figure S10. ^{13}C NMR of compound 3

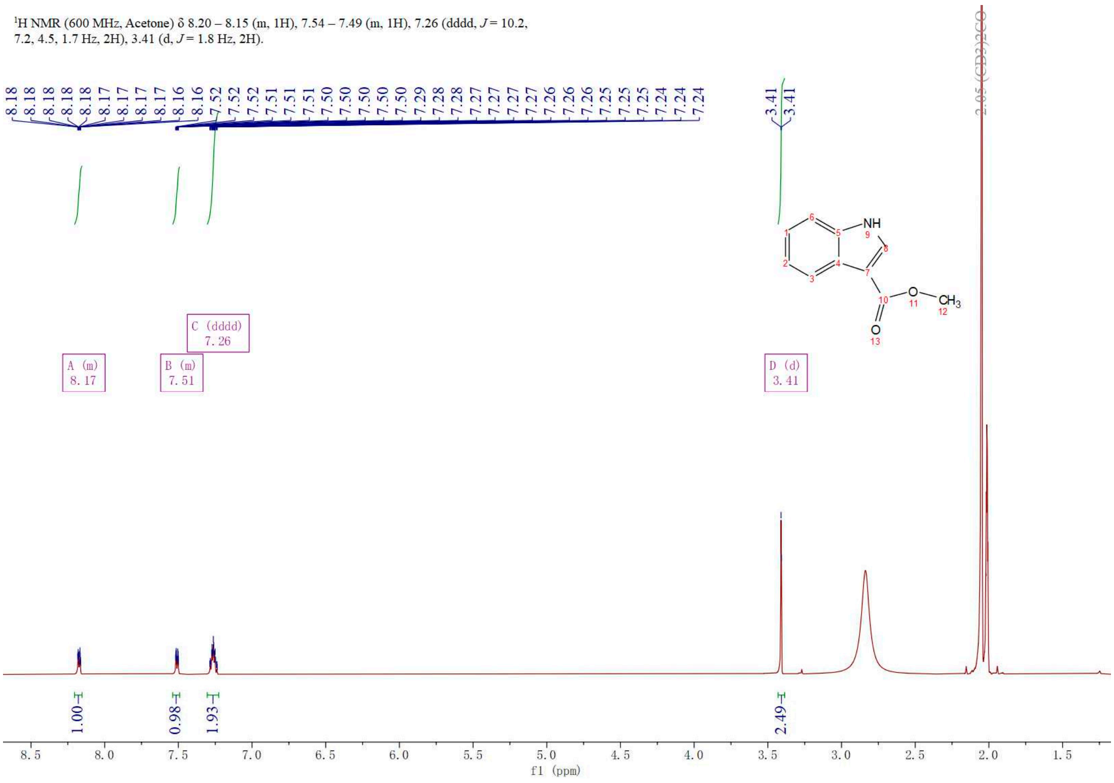

Figure S11. ^{1}H NMR of compound 4

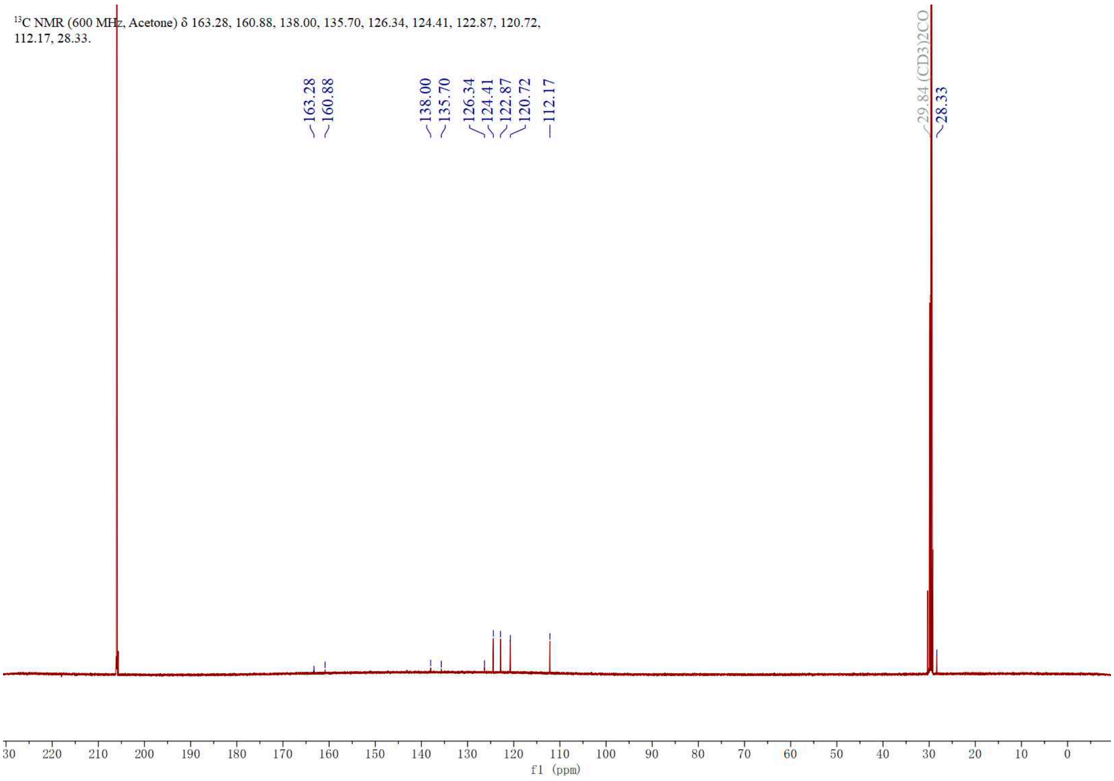

Figure S12. ^{13}C NMR of compound 4

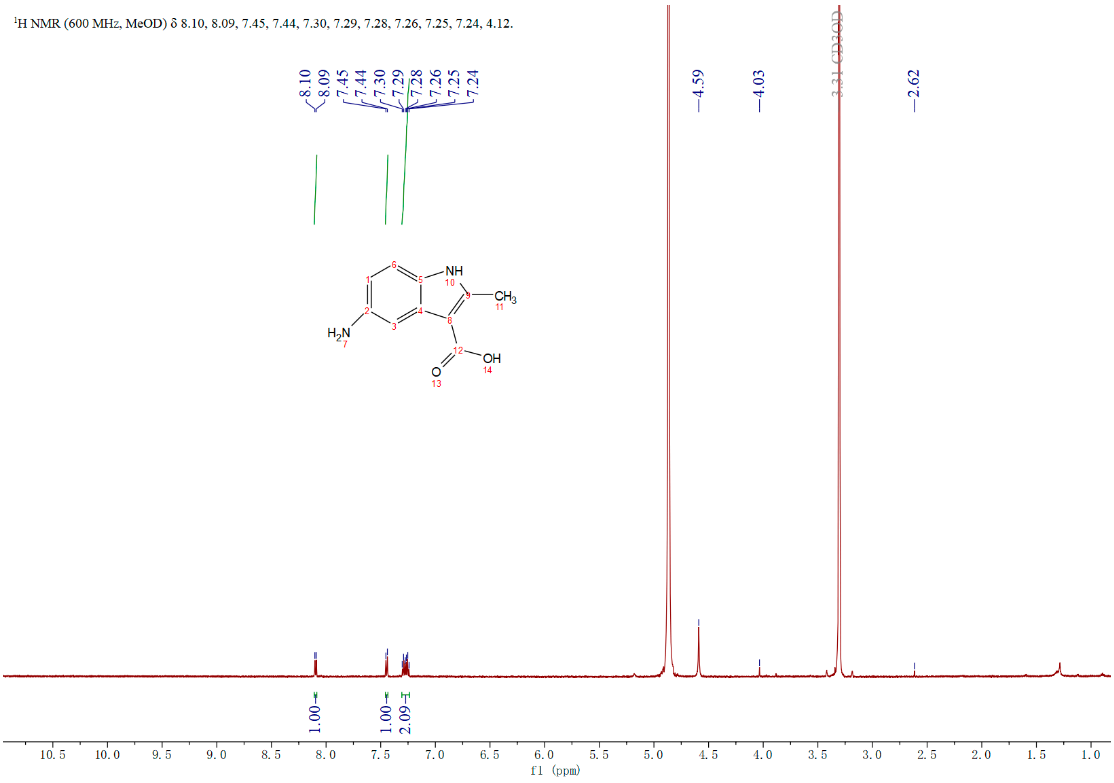

Figure S13. ^{1}H NMR of compound 5

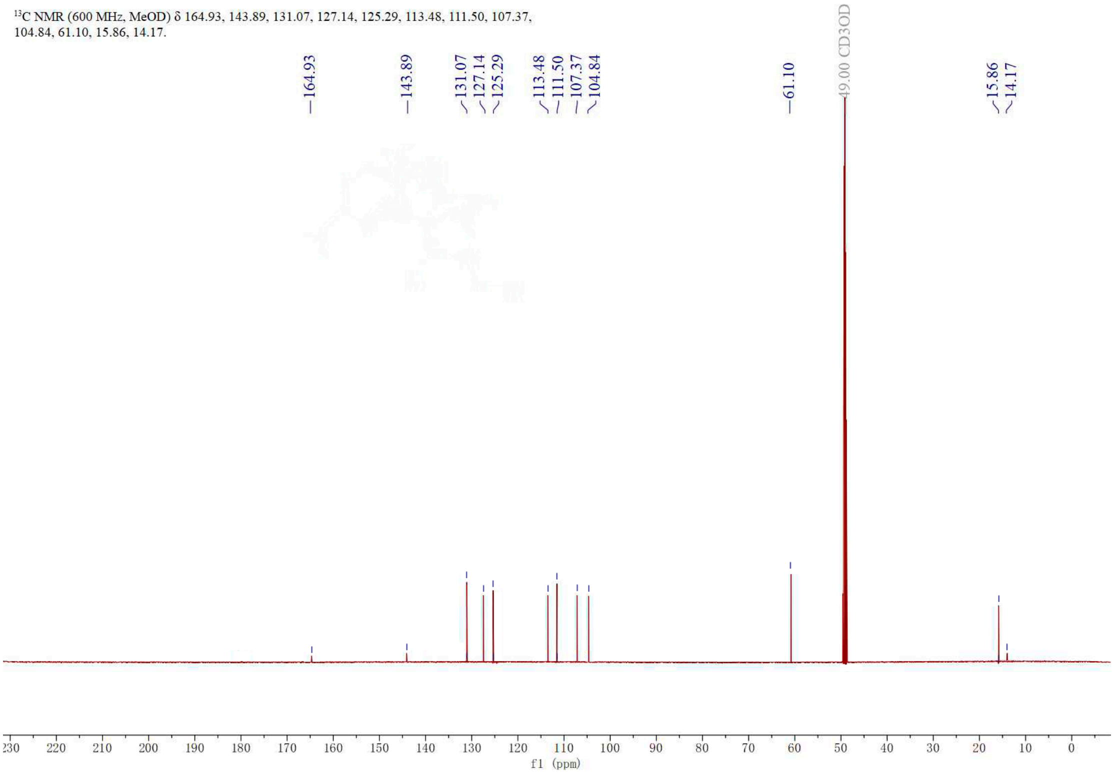

Figure S14. ^{13}C NMR of compound 5

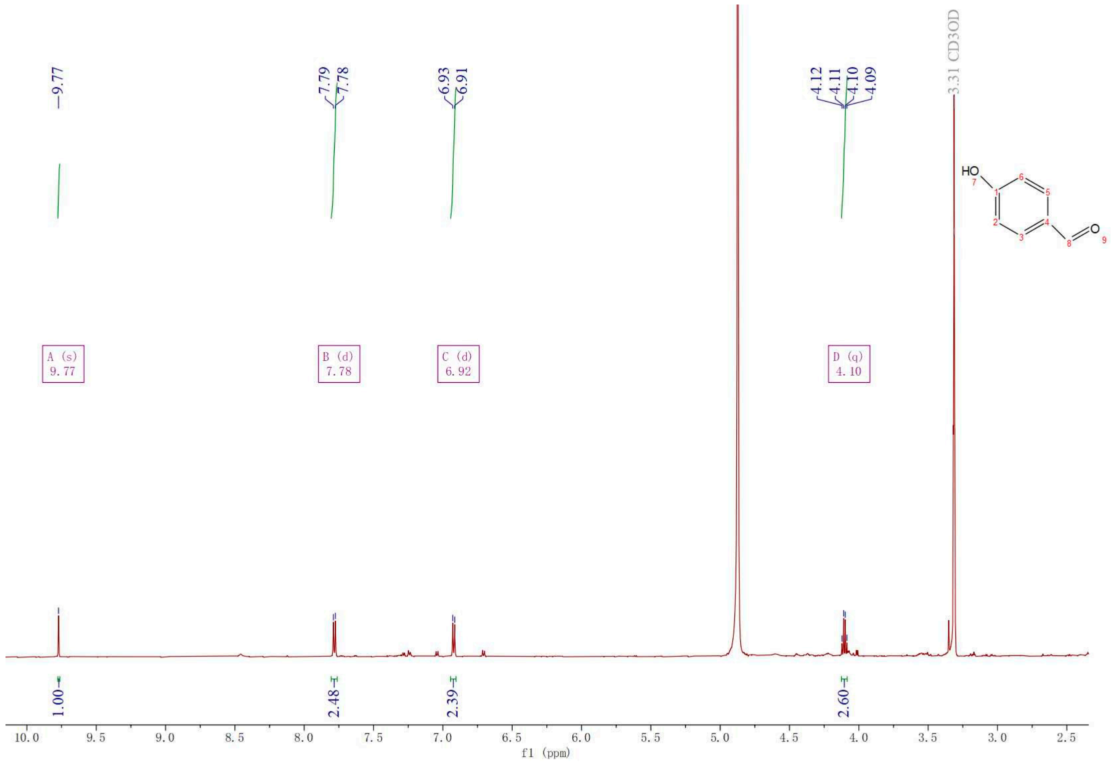

Figure S15. ^{1}H NMR of compound 6

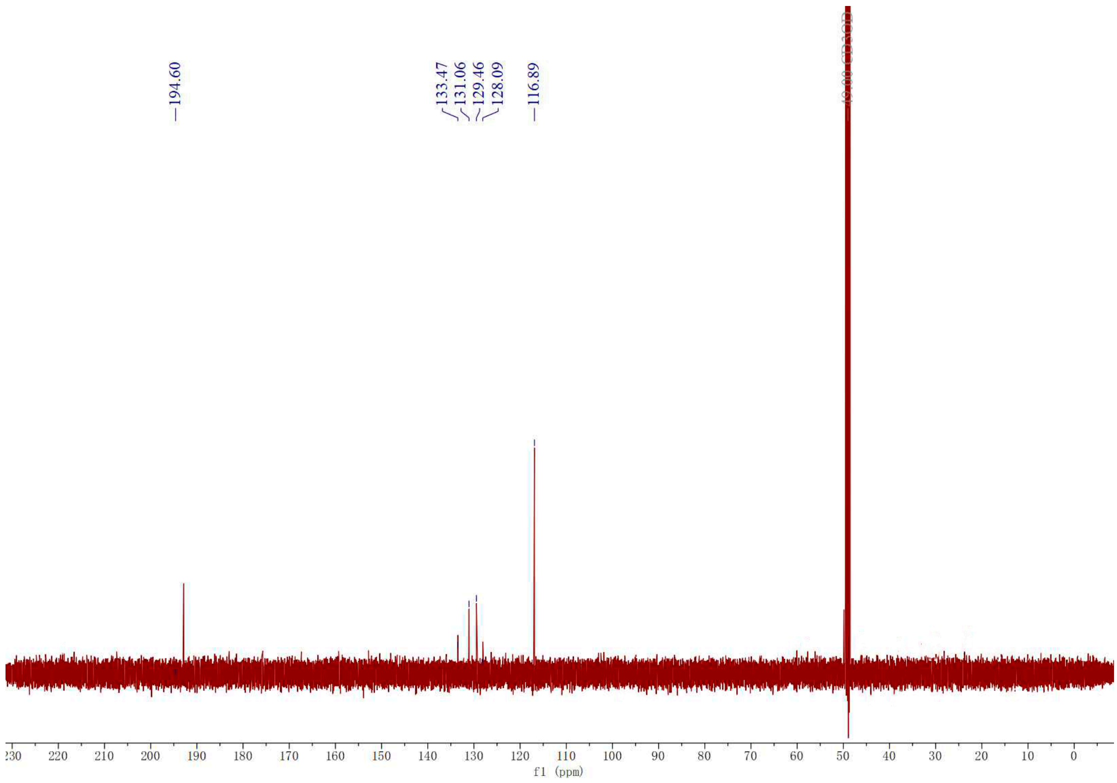

Figure S16. ^{13}C NMR of compound 6

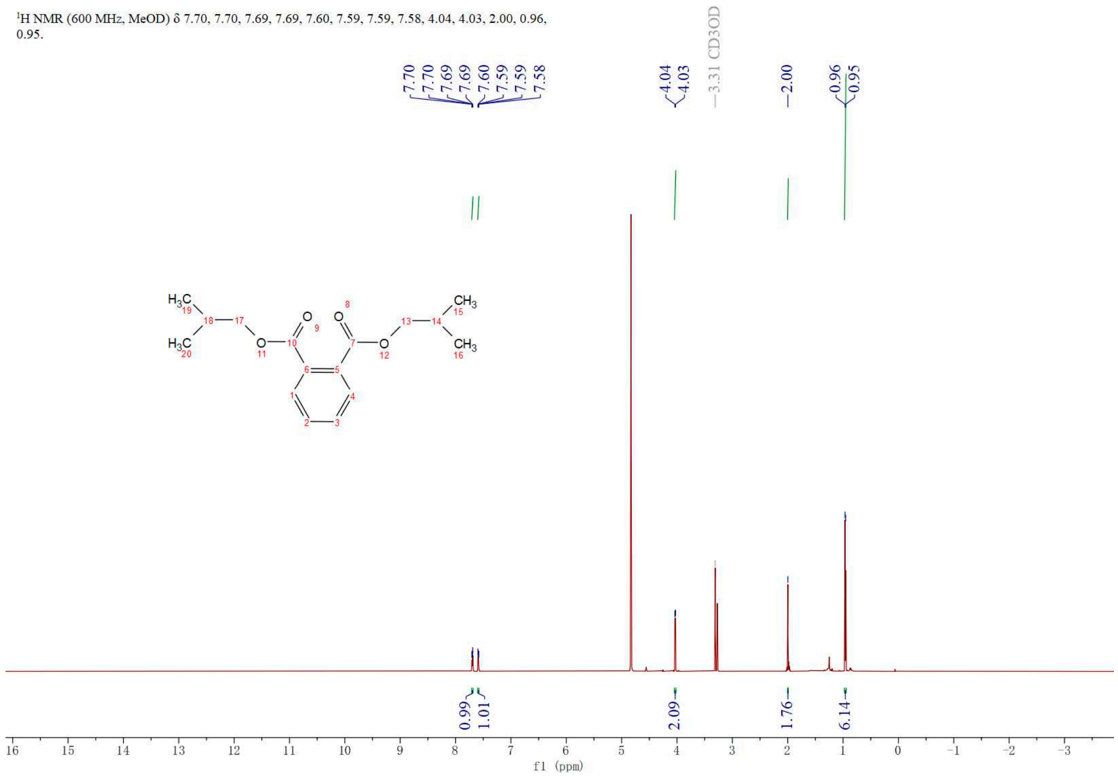

Figure S17. ^{1}H NMR of compound 7

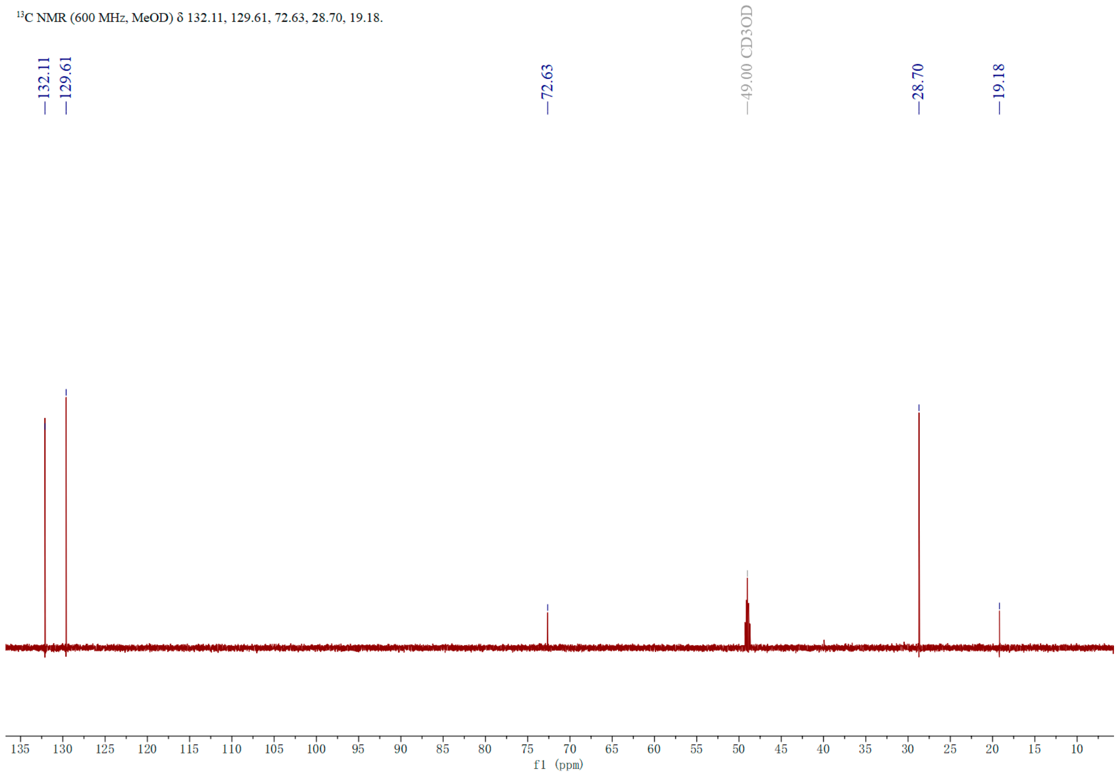

Figure S18. ^{13}C NMR of compound 7

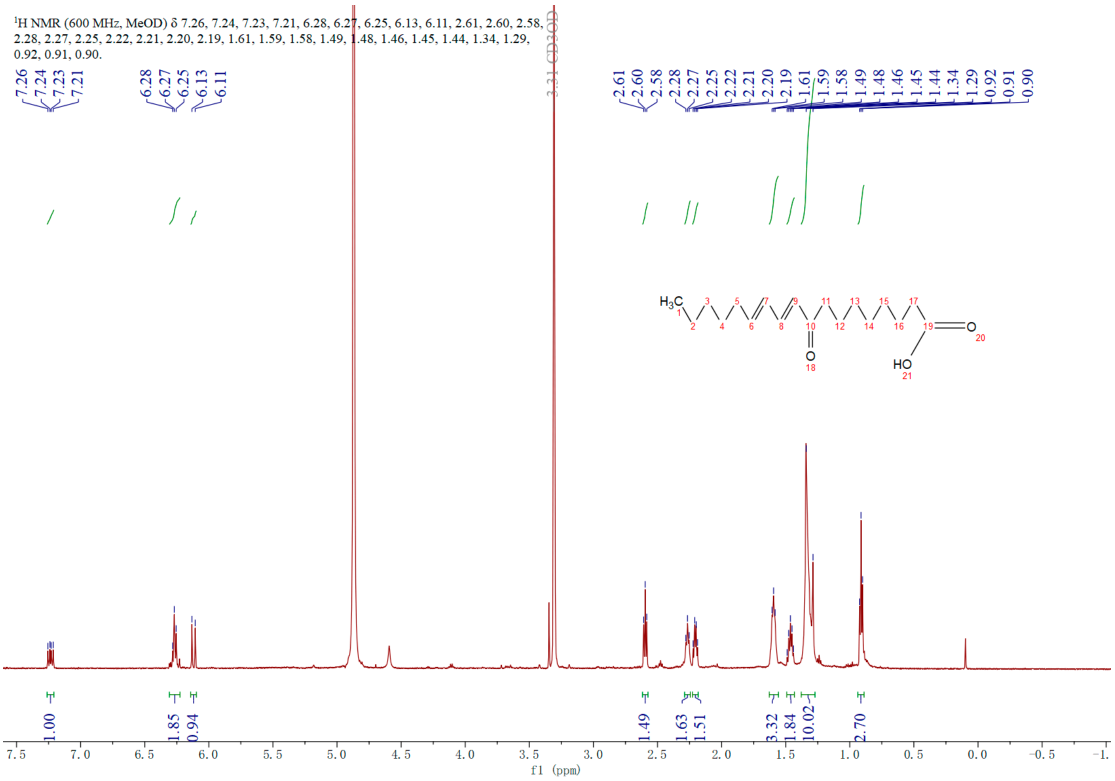

Figure S19. ^{1}H NMR of compound 8

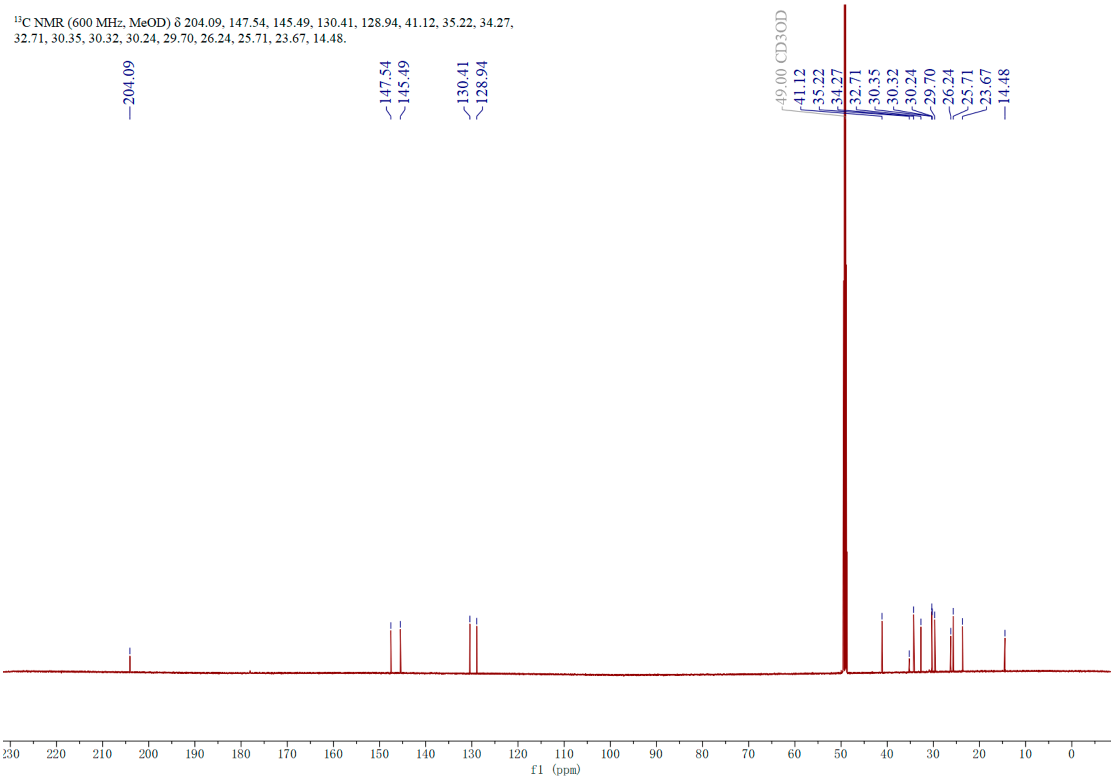

Figure S20. ^{13}C NMR of compound 8

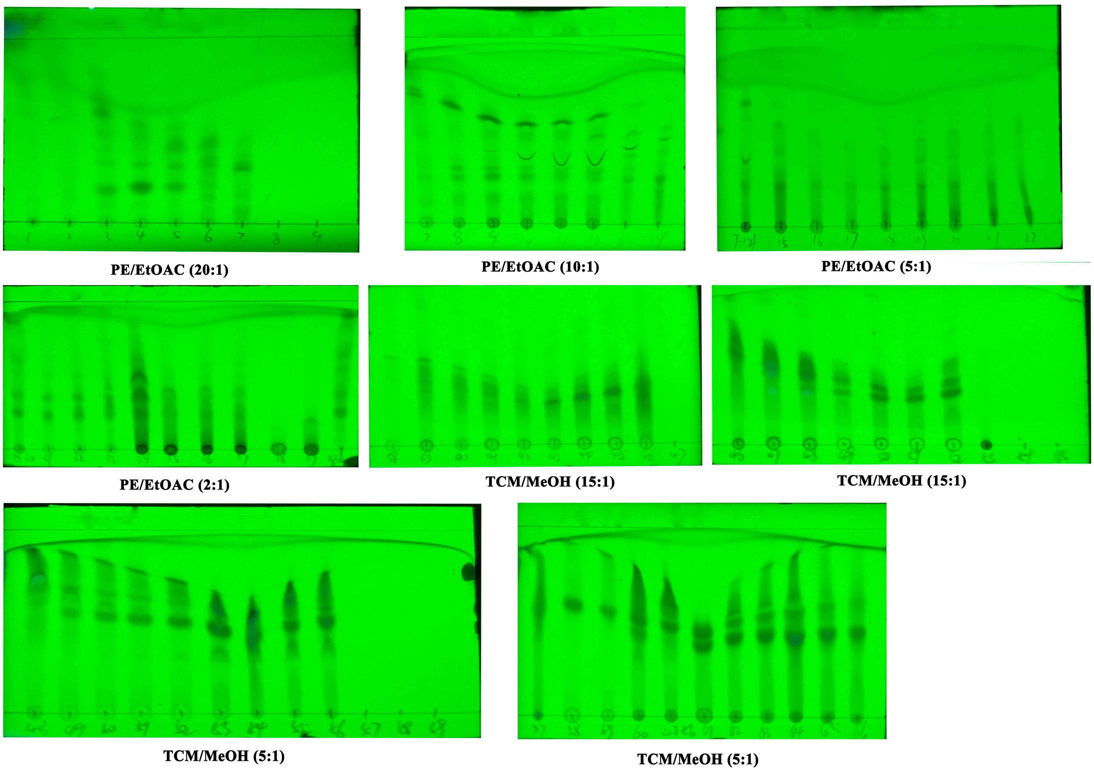

Figure S21. Thin Layer Chromatography (TLC) chromatogram of fractions

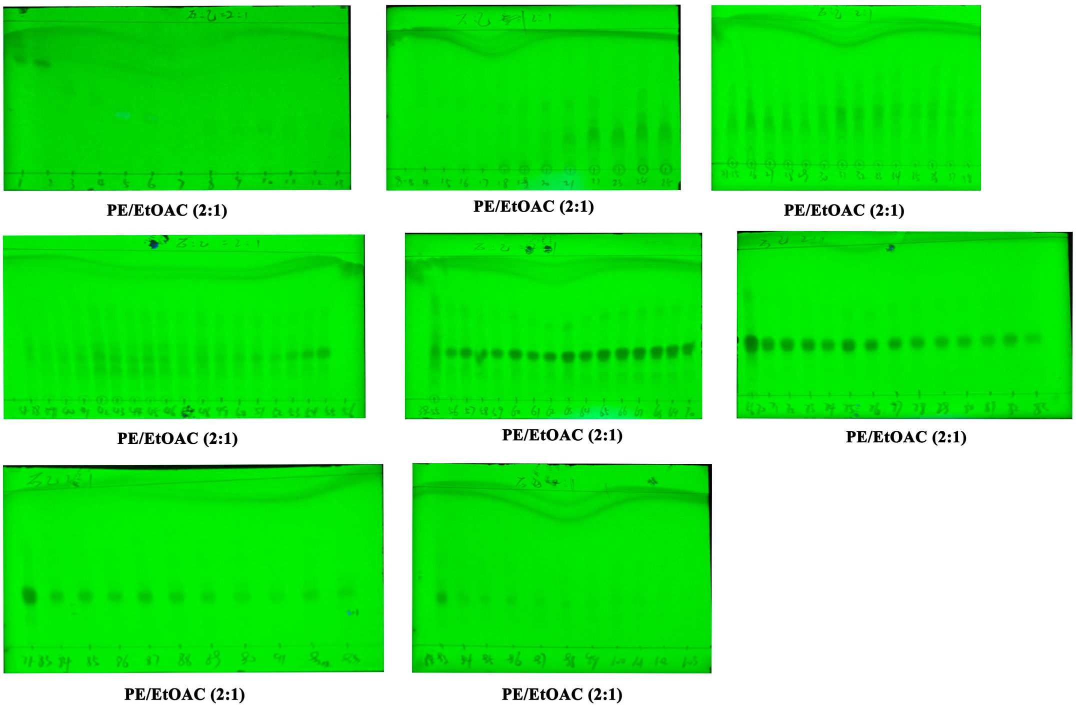

Figure S21. Thin Layer Chromatography (TLC) chromatogram of subfractions
